# Extra Supplement—The Nursing Mirror

**Published:** 1889-05-04

**Authors:** 


[*Extra Supplement.*

### The Nursing Mirror.

All Contributions for this Supplement should be addressed "The Nursing Mirror," care of the Editor, 139-140, Salisbury Court, Fleet Street, London, E.C.

### En Passant.

COMBINATION.—The number of answers received to the proposal to hold a meeting to consider how private nurses could best combine is ridiculously small. About a dozen post-cards and half-a-dozen letters conclusively prove that the desire for combination is far from being widely spread, and that probably most institutions are managed in such a way as to give satisfaction to the nurses who work for them. Under these circumstances we shall not, of course, take the trouble to make further arrangements in the matter, and, on the whole, we are glad to find there are so few nurses who are dissatisfied with their lot. "Fair Play," who had arranged a scheme for starting a home where nurses should take their own earnings, has not been able to find twenty companions to combine with her. Another nurse, "M. E. M. W.," who found life in an institution trying, and far from lucrative, took rooms in the neighbourhood in which she was known, and after paying for her room, which she keeps on while at her cases, finds herself far better off and far more comfortable than while attached to an institution. But, then, "M. E. M.W." was asked by the doctors to remain where they could procure her services. A thoroughly-trained nurse should always be able to command fair terms, and in the worst managed institutions we fear the nurses are not properly trained, and therefore cannot expect such high pay. It is a pity some of our best training schools send out nurses under two guineas a week; except in nursing for charity the charge ought never to be less than two guineas for a fully qualified nurse.

PROBATIONERS AND THE PRESS.—The article on "White Slavery in Hospitals" was the culminating point of exaggeration in the opinion of the public press on the duties of nurses; we may now hope for a cessation of much of the rubbish which has been written lately. The return to sense can be traced in an article on "Probationers," in last week's *The Lady*, which is undoubtedly written by one well acquainted with the subject in hand. The different styles of pros.—the patient, the flirting, the sensational, the supercilious, etc.—are each sketched in a few happy phrases which put before us word-portraits we can all recognise. Take this description of the silly pro., for instance, which is true to the very life: "The silly pro. is a special affliction to her neighbours, who cannot in the least imagine what brought such a helpless creature into such a position. Probably a pretty, affectionate child elsewhere, she is, without doubt, in the wrong place now. She leaves everything lying about, never shuts a door or closes a drawer; she tries to carry two or three jugs at once, and leaves a trail of milk-drops behind her for someone else to clear up; her aprons do not fit, her cap is crumpled, her dress too long; she is plaintive over her breakages and tearful over her blunders, and is alternately blamed and pitied, till her guardians wisely withdraw her from the scene of her failures. When the silly pro. returns to pay a friendly visit to her former companions, they are pleasantly surprised to find how much better she looks when differently clothed, and how rational she is in everyday life. It is noticeable that a true nurse always looks best in her uniform, and the young lady amateur always looks best out of hers." If any probationers have the courage to desire to see themselves as others see them, we advise them to read this article. Those who are contemplating becoming probationers might also read it with advantage to themselves and to others.

NURSE AND DRESSER.—The following conversation actually took place in the out-patients' department of a large hospital lately. Nurse: "Have you bandaged up that leg I asked you to do for me, Mr. B.?" Student (of three weeks' experience, innocently): "No; I held the leg, and the patient bandaged it, nurse."

BRITISH NURSES' ASSOCIATION.—The quarterly meeting of this association was held lately, when few members were present. Dr. Bedford Fenwick explained different parts of the proposed charter, and Miss Wood read a report of past progress. Male nurses are to be admitted as members of the association, which hopes, by the aid of drawing-room meetings and appeals, to push its projects. The next lecture will be by Dr. Cullingworth, on "Obstetric Nursing," on May 17th.

A HINT FOR MIDWIVES.—In the Jessop Hospital for Women at Sheffield there has not been a single case of ophthalmia amongst the last 2,000 babies. This is believed to be solely the result of training the nurses to wash the lids of the babies' eyes with tepid water as soon as ever it is possible to get at them. Care must be exercised not to open the lids, and after cleanliness must be attended to. It is estimated that more than 30 per cent. of cases of blindness are caused because these simple precautions are not carried out.

TRAINING AT NORWICH. — The Committee of Management of Norwich Hospital have seriously undertaken the thorough training of nurses. During the past quarter the systematic training of the nurses has been arranged by the honorary medical staff, by whom regular lectures are given in the elementary branches of anatomy and physiology, and in medical and surgical nursing. It is proposed to hold periodical examinations, and to grant certificates to those nurses who satisfy the medical staff at the final examination and of whom the lady superintendent may report favourably. These certificates will be signed by the chairman of the Board of Management, two members of the honorary medical staff, and the lady superintendent. It is hoped that the advantage thus offered will be deemed a full equivalent for such services as probationers are capable of affording, and that the institution will therefore be spared in future any charge for salaries to this class of nurses.

SHORT ITEMS.—The report of the Radcliffe Infirmary, Oxford, states that the system of probationer nurses continues to act well, and with the employment of nurses in private nursing when not wanted in the hospital, is of considerable assistance to the revenue.—The committee of Erith Cottage Hospital expressed at their annual meeting their appreciation of the unremitting care and attention of Nurse Homewood.—Sir Orfeur Cavanagh, in opening the Tolworth Isolation Hospital, mentioned the excellent qualifications of the matron in order to give confidence to medical men who desired to send private patients to the hospital.—Mrs. Barber, matron of St. Alban's Hospital, has been seriously ill, but is now recovering.—At the opening of the Lees Annexe of Oldham Infirmary, Dr. Thomson stated that nursing had now arrived at the dignity of a profession, and it behoved hospital authorities to give attention to the wants and comforts of nurses. The annexe provides accommodation for the nursing staff.—The annual report of the Pembrokeshire Hospital states that the nursing and dietary arrangements of the house are of a very satisfactory character, under the able management of the matron, Miss Bull.—The medical staff of Norwich Hospital have sent a nurse to Queen's-square to be trained in massage and electricity.

### The Nursing and Management of the Insane.

By T. Duncan Greenlees, M.B. Edin.

*(Being introductory to a course of lectures to the Nurses of the City of London Lunatic Asylum.)*

*(Concluded from page x.)*

It is very necessary that you should enter upon your duties unbiassed by any sentimental ideas; and I would impress upon you the importance of always recognising insanity as a disease, not necessarily of the morals or mind, but as an actual physical malady. The disturbances of thought, the distorted workings of the mental faculties, and the peculiarities of speech, actions, and manner, are merely the symptoms of disease in the same way as coughing is a symptom of bronchitis. When you learn to recognise the nature of insanity you will treat all the symptoms I have described with kindness, avoiding all resentment at what may appear to you responsible conduct by your patients.

To gain the confidence of your charges should be your first aim. Your manner should be frank and open, free from all embarrassment, and as natural as if you were dealing with sane people. Never show fear; cool and decisive conduct and self-possession inspire wholesome respect, and will help you out of many difficulties.

You should never ridicule or jest with your patients on their delusions; the practice of encouraging patients to rehearse their strange fancies and ideas for your own amusement, or for the entertainment of visitors, is to be condemned most strongly. The degree of slavery to which insanity reduces our fellow-creatures is such that insane persons, however amusing they may seem to be, are more to be pitied and sympathised with than laughed at. Ridiculing patients about their delusions often tends to strengthen their hold upon their minds, and thus the ultimate chances of recovery are correspondingly diminished.

There is a great deal to be learned in the wards of an asylum; by careful observation you will get to know much of your patients, their temperaments, their modes of thought, and the various mental and physical characteristics of each individual under your care. The noblest study in nature is man, and the most interesting study of man is his mental and moral attributes. If you devote your energies to such a study, the experiences you will thereby obtain will aid you in dealing with your patients in times of emergency, and will be of great assistance to you in the management of new patients as they are, from time to time, brought under your care.

The habit of considering the management of insane patients, as similar to that made use of in children, is the ground work of a sensible mode of treatment. As we deal with children, directing, controlling, and leading them into right ways of thought and action, so should we also deal with the insane. To oppose violence with violence is utterly irrational; don't let your forbearance give way to anger or petulance, nor allow pity and sympathy for your patients to be forgotten in a moment of irritation. The qualities of the heart are as essential as those of the head, and a nurse can only attain perfection by a happy blending of the two. Everything in your power should be done to make confinement in an asylum as free from discomfort and restraint as possible, for the deprivation of liberty is the hardest burden most of our patients have to submit to.

The work of caring for and nursing the insane is arduous, very responsible, and not unaccompanied by personal danger; the hours of duty are long, there is much to contend with at all times, and the immediate associations of the insane are in many cases by no means pleasant; yet, with all its discomforts, I believe the work is such as to develop in you all that is best and noblest, and your character should improve under its influence, since it calls for the devotion of your lives for the sake of your afflicted brethren.

I am anxious that, instead of being merely the keeper of your patients, the gaoler who prides himself in his possession of the keys of liberty, you may become their friend, companion, and nurse, and that your good example may always be before them as an encouragement to good behaviour, and, it may be, a stimulus to arouse them from their morbid, dormant mental condition.

Discharge your duties in an unselfish spirit, and with cheerfulness, remembering the good old adage, "Whatever is worth doing at all is worth doing well," holds good in asylums as well as in other places. You may receive little encouragement from your patients, but never forget that the little seeds of kindness, sown around you day by day, may in time spring up to deeds of gratefulness; and, if things sometimes look dark and hopeless to your despairing eyes, recollect that you should

> "Be content in work
> To do the thing you can, and not presume
> To fret because it's little."

### Nursing in the Soudan.

Part IV.—Nursing at Sea.

Our thoughts now turned with special interest to the "Iberia." A day or two elapsed, however, before she could be cleaned and made ready for her new passengers. The strong and lusty had left her; the halt and maimed were to take their places. Very soon our aid was called for, and we were busy doing our part in turning the "Iberia" into a hospital ship for 102 sick and wounded officers and men. The two decks amidships were hung with hammocks, about six abreast, with a good space between, to allow for oscillation and the easy passage of attendants. On each deck there were some 40 hammocks.

The sick officers were in the cabins used ordinarily for saloon passengers, which were made as comfortable as circumstances would allow. The berths in each cabin, with the exception of the one in use, were taken away, thus enabling the occupant to have as much air and space as possible. Still, with all this thoughtfulness for their comfort, the cabin of a ship was but an indifferent sick-chamber. This formed the hospital. In other parts of the ship were accommodation for large numbers of convalescent men, the dispensary, hospital stores of every description, and all the other necessaries for people equal in number to the inhabitants of a good-sized village. It would be impossible to speak too highly of the organisation in every department of the hospital ship—all thought of, nothing forgotten.

It was really wonderful how the large amount of supplies could be measured so accurately to last the time required. Of course, economy was the watchword, and had to be adhered to strictly. None of the waste so common in large hospitals, where, without thought, tow and lint are too often put to other than legitimate purposes, could be allowed. The hours of work and recreation were arranged for the sisters; they went on duty at 7 a.m, taking temperatures, feeding helpless patients, and assisting the orderlies with especially bad cases. At 8 a.m. came breakfast, and at 9 the medical officers' rounds, which took a long time, the dressings often not being finished until noon; for, bear in mind, we were on the sea, our hammock-beds swinging hither and thither with every movement of the waves, and you have some idea of the difficulty of keeping ourselves and patients sufficiently steady.

A lurch of the vessel would upset the equilibrium of everybody, and over everything would go—lotion, dressings, doctor, sister, and all; but the calamity was usually borne by the patient with the stoicism born of a soldier's life. Perhaps, among the most awkward things to deal with were fractures of the lower extremities, and very funny were some of the devices for effectually putting in position the necessary splints, fracture boards, and extension. Officers and men alike were splendid fellows. No grumbling or murmuring was ever heard, but the best was made of everything cheerily and bravely, and this, remember, in spite of terrible wounds from spear, sword, and gun, and weary bodily illness. To tend and nurse them was work many might envy. Under the circumstances, all operations, whether of much moment or not, were difficult, and it was wonderful how cleverly they were performed by the medical officers. Cases of hæmorrhage were not infrequent, and from amongst the wonderful stores ice was at once forthcoming. The great heat as we steamed up the Red Sea was very trying to our patients.

Easter Sunday found us in the Bay of Suez, in a storm so violent that for hours we were at anchor, waiting for the Custom House officers, who could not come on board until it abated. At last we were free, but too late to enter the Canal that evening, and we lay at anchor all night, intending to start at daybreak. At Suez we received letters and newspapers; the latter contained news from England, of the engagement with the enemy on Sunday, March 22, under Sir John M'Niel. All our wounded on board were sufferers from that disastrous engagement, and most eager were they to hear what the home papers had to say about it. The number of papers was limited ; we therefore decided to read aloud, that all the men might hear, one and all being eager for the news. And as we were in the quiet waters of the canal, this was an easier matter than would otherwise have been the case. Most entertaining were their remarks as the different deeds of bravery and pluck were recounted. "I say, Bill, that was you." "Jack, do you remember those black fellows gave it us pretty hot there, didn't they?" "Ah, that was where I got this spear wound!" "Sister, please read that again," etc., were their running comments, as the reading proceeded. Some of the officers on board had been badly wounded in the same engagement, and most touching were the enquiries made by the men for their officers, showing what kindly feeling existed between them. Alas! among so many seriously ill, it was most unlikely we should get to England without losing some. Soon after we left Port Said, one patient who had been hopelessly ill with dysentery succumbed. And a few hours later another, with terrible spear wounds in the back, died. Funerals at sea are, perhaps, of sad sights the saddest, and nothing but the thought of the immortal spirit in man makes it bearable. It is usual to stop the engines, and toll the bell, while all who can assemble for the sad ceremony. In this case the commanding officer read the burial service, and in a few seconds all was over. Of our number we only lost four during the voyage home, and they were cases almost hopeless from the first. Reading aloud to the men proved so satisfactory to them, that we were called upon to repeat the experiment whenever we could. Sometimes it was almost impossible to make ourselves heard above the noise which is the inevitable accompaniment of a steamer in rough weather, to say nothing of being able to keep our feet; but we did our best to entertain them and while away the many weary hours.

Most of the orderlies were good nurses, and were kind and attentive to their comrades. Our number of sisters was too limited to allow of much supervision or nursing during the night; our efforts were confined to late and early rounds, and an occasional nocturnal visit to a few specially bad cases. When the weather was fine and warm, those of our patients who could bear it were taken on deck. This was a delightful change for them from the hot and stuffy lower decks; but not many days, however, were warm enough for the majority of cases. So our journey progressed, the daily work occupying fully our time and thoughts.

The men, poor fellows, made anxious enquiries as to "where we were," and great were the rejoicings when "Gib." and the "Bay" were passed. The latter, usually so unpleasant, was on this voyage calm and peaceful, which, for our patients' sake, if not for our own, was much appreciated. We left Suakim on the 2nd April, and on the 19th Portsmouth was in view. We had not lost much time *en route*. All were rejoicing at being home once more. April the 20th the disembarkation of our invalids took place; the morning was bright and warm, and all were astir early. The ship entered Portsmouth Harbour, where the train to take the men to Netley was alongside the quay, and the patients were carried on stretchers to the carriages. One or two very bad cases were sent by sea to Netley, that mode of travelling being thought less trying for them than by rail. Our orders were for the nursing staff to proceed at once to London and report ourselves to the authorities there, and we reluctantly took leave of our patients and other friends, and were soon off to town.

*(To be continued.)*

### Workhouse Infirmary Nursing Association.

By kind permission of the Hon. Mrs. J. G. Talbot a most interesting meeting of the Workhouse Infirmary Nursing Association was held on April 2 at 10, Great George-street. The Countess Brownlow, Lady Colebrooke, the Lady Wantage, Lady F. Cavendish, Lady E. Cavendish, Hon. Mrs. Hardcastle, Mrs. Bonham-Carter, and many others, besides several matrons of infirmaries. Mr. Rathbone, M.P., was unavoidably prevented from opening the proceedings, but his place was ably filled by Mr. Talbot, M.P. Miss L. Twining's exhaustive paper brought many important and, in some cases, startling facts to light. She dwelt upon the need of trained inspectors, particularly ladies, for our workhouse infirmaries, especially country ones, where even the absolute necessaries, such as lint, etc., are unknown. The same subject was still further enlarged upon by Canon Erskine Clarke, who pointed out the existing difficulty of dealing with patients whose cases, being too hopeless for an infirmary, are drafted on to the unions. In many cases he considered it possible that hospitals would receive the most acute cases if payment were made by the guardians. Then, after enlarging upon the inconsiderateness and mistaken kindness of untrained matrons, he gave place to Miss Wood, who, introducing herself as a "trained nurse," laid her valuable experience before the meeting. Nursing, she urged, was nursing, whether in hospital or union, and so far from trained nurses meaning heavier rates, this result would be obviated by the quicker and more thorough cure of the sick. In conclusion, she drew attention to the very large area of country as yet unprovided with hospitals or infirmaries. Dr. Spicer took for his theme one hitherto untouched on—viz., the proper care of the children. He pointed out how in many cases they are left entirely to pauper help, and often discharged half cured, to be returned again in two days' time. At the conclusion of the meeting, Mr. Rathbone, who had come in later in the evening, spoke of the immense good that had been done by trained women in the last 22 years, before which time nothing but pauper help was employed in our workhouse infirmaries, and after a vote of thanks, proposed and seconded, to Mr. and Mrs. Talbot, Miss L. Twining read aloud the yearly income of the association since its commencement. In only one year had it exceeded the sum of £300.

### Everybody's Opinion.

*[Correspondence on all subjects is invited. No communications can be entertained if the name and address of the correspondent is not given, or unless one side of the paper only be written on.]*

### RECREATION FOR NURSES.

MAJOR MEARS writes: I am most anxious to meet with friends who are interested in the matter to arrange a plan by which hospital nurses could have a couple of hours' driving or boating once or twice a week during the summer months, either early in the morning or in the afternoon. I have tried what I now suggest on a very small scale, with most excellent results; and nurses to whom I have mentioned the idea, without a single exception, thought it a happy one. Two hours in the fresh air, with a cup of tea, would send them back to their work all the better and fresher.

### LADY NURSES.

A. S. writes: If nurses were only chosen from those who must work or starve, we should not have so many complaints; far be it from me to put a stumbling-block in the way of any woman who wishes to learn how to be useful in a sick room, but real workers do not think of luxuries so much. I suppose because they have had hard times at home, and know there is nothing between hard work and starvation. If lady nurses do not find their food good and well cooked, why do they not complain at headquarters? plenty of good wholesome food, nicely served, means economy not extravagance. Every woman worthy the name of "matron" is quite able to arrange for the comfort and well being of her nurses, and will not be likely to forget her probationer days, which are generally very happy ones; but let no one for a moment submit to even the idea of a "body of philanthropic ladies" coming in to arrange the "off and on hours of nurses." To put it mildly, lady visitors are a great bother, and matrons have plenty of worries without adding to them.

### WHITE SLAVERY IN HOSPITALS.

A. C. writes: The article on "White Slavery in Hospitals," in the *Pall Mall Gazette* of April 3rd, is in the main very true, but with regard to the long hours the public is the proper horse to saddle. The real truth is hospitals are badly supported, are always more or less in debt, every penny has to do duty for two, consequently the nursing staff is kept to as small a number as possible Again, all women have to work hard; their work is never done, as not only nurses but mothers of families can tell, together with servants in lodging-houses. Most, if not all, matrons are trained nurses, and surely they have the well-being and comfort of their nurses at heart. The public requires a good deal of education before it will understand that nurses cannot strike; they are too poor, wages too low, too many ready to do the same work for less money, and so many ladies, who do not require to earn their living, have taken up nursing, and pushed many a poor sister further into the cold. By all means let ladies learn how to nurse, but let them pay a high premium. Early rising will hurt no one, but good food—with two hours off daily, half a day off once a fortnight—should be insisted on. The yearly holiday, three weeks or a month, divided during each six months, but there again comes in expense. Few can afford two journeys, and many have to pay a consideration for board at home, for nurses are often of a poor race. Some hospitals, doubtless, work their nurses much harder than others, and care less for their comfort. There, again, the public should provide a proper number of separate bedrooms for the nurses; after all day in a crowd, as it were, a little solitude is good. Food should be plentiful, and well cooked. There is no excuse for a badly supplied table. There, again, hospitals greatly differ, and there is no economy in stinting the food, or docking the nurse's wage when she is ill—shame to know it is done. Extravagance should be put down, but at best hospital work is very hard, and tries all, and even the H. S., who certainly has the best of it, gets done up in a few years. Therefore, all connected with the inner working of a hospital should expect rooms comfortably furnished, and well away from the noise and smell of wards. Many bedrooms are still over wards, or, worse still, leading out of wards. Sitting-rooms so close to wards that a little music after 8.30 p.m. is out of the question. No garden, only hot, dirty streets for tired women who, whether they take to nursing for the love of it or to earn their bread, as a rule, do their work patiently and well, and only those at work fourteen and fifteen hours a days in bad air, and amongst sick people, know the real meaning of "tired out."

THE FOOD QUESTION.

A Late Nurse writes: Nurse Eva Carter, in her letter of the 6th ult., wishes for opinions of other nurses, in reply to which I am sending my experience of eight months in a fever hospital under the Asylums Board. When I went there I was in excellent health, but, owing to the very indifferent manner the nurses were fed, I gradually began to fail. I may add, although the nurses in a body complained to the medical superintendent, no alteration was made, and we were told by him that the fare provided was all the Asylums Board allowed, also that we were not supposed to have *good* food! The arrangements were as follows: Breakfast at 6.30 a.m., bread and butter, the latter very inferior, with tea (at times all but cold); five days out of seven eggs were served boiled, which were anything but fresh. Nothing was provided between that but bread and cheese. Dinner at 2 p.m., which consisted of meat and potatoes; three days out of seven pork is served (it has appeared oftener); potatoes, in jackets, not always clean. Perhaps twice during the week a second vegetable appears in the way of greens, parsnips, or carrots, those very often uneatable. Bread and butter for tea all the year round. Then as to supper, the remainder from dinner, if any. It is a fact that both day and night nurses have gone to bed hungry. I myself spent the greater part of my salary in food and medicine.

A HOLIDAY HOME.

The Matron of a London Hospital writes: As the holiday time is now drawing so near, I feel sure many of your readers will be glad to hear of a pleasant home in which to spend them. I can from personal experience strongly recommend the home at 9, Connaught-road, Folkestone, the lady superintendent of which is Miss Orodwell, who does her utmost to make her visitors' holiday a pleasant one. The terms are very moderate, only 15s. weekly for board and apartments, in addition to which each visitor is entitled to a railway pass with which she can obtain a return ticket from London to Folkestone for 5s.

### Vaccination and its Opponents.

With reference to our note under the above heading, on page xi. of "The Nursing Mirror" for 20th ult., a Public Vaccinator writes:

About a fortnight since I was fulfilling my duties as public vaccinator at my surgery when a woman brought one of her grandchildren to be vaccinated. While I was performing the operation she casually remarked that "her other daughter took her child to be vaccinated to Mr. ——." I replied "How was that?" and the answer I got was that "Mr. —— was not so particular as I was." This, of course, can be read two ways, but the way she intended me to interpret it was that Mr. —— was not so particular about the places standing, their number, or their size. I think this applies remarkably to the article in last week's *Hospital*, and I must say I agree with what is stated. At all events, I think that only public vaccinators ought to be allowed to give certificates of unfitness for successful vaccination for more reasons than one; but more especially because they are supposed to be responsible for the good vaccination of their districts.

### Lectures.

On Thursday, May 2, and five following Thursdays, at 5 p.m., in the Lecture-room, Zoological Gardens, Mr. F. E. Beddard will lecture on "Some of the Interesting Animals in the Gardens." Fee for the course, 5s.

On May 8, 15, 22, 29, and June 5 and 12, lectures on "Archæology" will be delivered at University College. Prof. R. S. Poole will inaugurate the series. Tickets for the course can be had (free) from the secretary of the college.

On May 21, 28, and June 4 and 11, Prof. Ray Lankester will lecture on "Some Recent Biological Discoveries," at the Royal Institution, at 3 p.m. Fee for the course, 10s. 6d.

Bolton Infirmary.—The Matron of the Infirmary and Dispensary, Bolton, writes to say that the post of sister for female wards is no longer open. She daily receives applications, and regrets it is not possible to reply to them all.

### Appointments.

Easter Ross Poorhouse.—The House Committee have appointed Miss Lizzie Gloag as charge-nurse of this union infirmary, near Tain, N.B. Miss Cloag trained at the Dundee Infirmary as a lady probationer under Dr. McCosh and Miss Lebrun, matron, and practised at Glasgow Royal Infirmary and in private nursing. The post to which she is now appointed is an important one, and we congratulate the committee on having selected a trained nurse of ability.

German Hospital, Zanzibar.—Countess Asta Blucher and Frau von Borke, of Berlin, have been appointed superintendents of nursing at the Hospital of Invalids belonging to the German Navy and Captain Wissman's force. The appointments are made by the Women's Association, and the Countess and Frau von Borke will sail immediately for Zanzibar.

### Vacancies.

The following vacancies are announced:

**Matron.**

Manchester and Salford Sick Poor Institution, £60.

**Sisters.**

Pendlebury Hospital, £30.
Bolton Infirmary, £25.

**Nurses.**

Shepton Mallett Hospital, £30.

Birmingham Infirmary, £25.

Carmarthenshire Infirmary, £20.

Kendal Hospital (night), £28.

Newport Infirmary.

Poplar Hospital (night), £20.

Jersey Institute, £25.

Eltham Cottage Hospital.

Torquay Institution, £22.

West Kent Institution.

Birmingham Fever Hospital.

Eastbourne Nursing Institution.

Leicester Institution.

St. Cuthbert's Poor House, £25.

Kingston Home, Hull.

Rochdale Infirmary, £22.

Royal Hospital for Children and Women.

Royal Hospital for Diseases of the Chest.

Ventnor Consumptive Hospital £20 to £25.

Holborn Union (two night, two assistant), £20 and £17.

Berry Wood Asylum, £25 to £30.

Hampstead Home Hospital (several) £20.

Royal Surrey County Hospital (assistant), £16.

West Sussex Infirmary (two), £30.

**District Nurse.**

Eaglescliff, Yarmouth, £60.

**Probationers.**

Wrexham Infirmary (two).

Halifax Infirmary.

West Herts. Infirmary, £10.

Burton-on-Trent Nursing Institution.

Buckinghamshire Infirmary.

### Notes and Queries.

**Queries.**

(141) *Papier Goudronne.*—Where can this be obtained? *Amateur Nurse.*

(5) *Red Pepper and Cold Packs.*—How should these be given?—*Nurse Norah.*

(6) To whom should I apply for information concerning Lady Dufferin's Fund?—*Agnes.*

(7) *Printer of Certificates.*—Matron again appeals to the courtesy of her fellow-workers to give the address of a printer of certificates for nurses. Many must know such an address, and might take the trouble to send it.

(8) *Nursing in America.*—I should be glad of the address of some training schools in New York or neighbourhood. *A.*

(9) *Accoucheuse.*—Can anyone tell me of a town or suburb in which a doubly qualified midwife and monthly nurse (lady) could earn a certain income of not less than £120 to £150 per annum, and where her daughter could meet with employment as a daily governess? Both have good testimonials; or is there any such opening in the Colonies? Canada or United States preferred.

(10) *Cellular Clothing.*—Is the cellular clothing, which was mentioned in *The Hospital* some time ago, still manufactured, and on sale? And where can it be bought?

**Answers.**

(138) *Nursing in France.* –Apply to Miss Pearson, Les Agaves, Cannes, or to Miss Woodcock, Holland Institute, Nice, or the Home for Invalid Ladies, San Remo.

(5) *Red Pepper and Cold Packs.*—For the first, put a teaspoonful of red pepper in a pint of tepid water, soak a flannel in it, and wind it round the patient's body. For the second, place the patient in a bath of 80 degs. Fahr., and gradually reduce the temperature to the degree ordered, or else wring out cloths in water of the temperature ordered and wrap the patient in them, changing them constantly.

(6) *Lady Dufferin's Fund.*—Apply to the Hon. Secretary of the United Kingdom Branch of the National Association for supplying Female Medical Aid to the Women of India, 65, Kensington-gardens-square, W.

"*Work To Do.*"—The "London Diocesan Magazine" is published by Messrs. Griffith and Farran, St. Paul's-churchyard. Price 2d. We cannot answer so many queries privately when there is no necessity for doing so.

*Lady Roberts' Homes.*—All information concerning these homes can be found on page 163, Vol. III. of *The Hospital*, where there is an article by Lady Roberts. The scheme is in no way connected with Lady Dufferin's Fund, and is for supplying homes in the hills where nurses can be moved during the hot season. Such a home is open at Murree.

